# Alcohol, Tobacco and Illicit Drug Use During Pregnancy in the Longitudinal BELpREG Cohort in Belgium Between 2022 and 2024

**DOI:** 10.3390/jcm14020613

**Published:** 2025-01-18

**Authors:** Sien Lenie, Laure Sillis, Karel Allegaert, Annick Bogaerts, Anne Smits, Kristel Van Calsteren, Jan Y. Verbakel, Veerle Foulon, Michael Ceulemans

**Affiliations:** 1 Clinical Pharmacology and Pharmacotherapy, Department of Pharmaceutical and Pharmacological Sciences, KU Leuven, 3000 Leuven, Belgium; sien.lenie@kuleuven.be (S.L.); laure.sillis@kuleuven.be (L.S.); karel.allegaert@kuleuven.be (K.A.); veerle.foulon@kuleuven.be (V.F.); 2 Research Foundation Flanders, 1000 Brussels, Belgium; 3 L-C&Y, Child and Youth Institute KU Leuven, 3000 Leuven, Belgium; annick.bogaerts@kuleuven.be (A.B.); anne.smits@uzleuven.be (A.S.); 4 Department of Development and Regeneration, KU Leuven, 3000 Leuven, Belgium; kristel.vancalsteren@uzleuven.be; 5 Department of Hospital Pharmacy, Erasmus MC, 3015 GD Rotterdam, The Netherlands; 6 Faculty of Health, University of Plymouth, Devon PL4 8AA, UK; 7 Neonatal Intensive Care Unit, University Hospitals Leuven, 3000 Leuven, Belgium; 8 Department of Obstetrics and Gynaecology, University Hospitals Leuven, 3000 Leuven, Belgium; 9 Department of Public Health and Primary Care, KU Leuven, 3000 Leuven, Belgium; jan.verbakel@kuleuven.be; 10 Nuffield Department of Primary Care Health Sciences, University of Oxford, Oxford OX2 6GG, UK

**Keywords:** pregnancy, substance use, preconception care, observational research, Belgium

## Abstract

**Background/Objectives:** Substance use during pregnancy is associated with adverse outcomes for both mother and child. This study aimed to determine the prevalence and determinants of alcohol, tobacco and illicit drug use before and during pregnancy in Belgium. **Methods:** An observational study was conducted using data from the longitudinal BELpREG registry. The study included women aged 18 years or older who completed at least one questionnaire on substance use during pregnancy between 2022 and 2024. Data were analyzed using descriptive statistics and logistic regressions. **Results:** In total, 1441 women were included. Preconception prevalences of alcohol, tobacco and illicit drug use were 82.2%, 10.0% and 4.2%. These self-reported prevalences dropped in the first trimester to 12.9%, 4.1% and 0.6%, respectively. Considering the rates of substance use in pregnancy but before pregnancy awareness, the overall prevalence of alcohol, tobacco and illicit drug use in the first trimester was 41.0%, 6.6% and 1.2%, respectively. Women with a higher education (aOR (adjusted odds ratio), 2.12; 95% CI (confidence interval): 1.14–3.96), unplanned pregnancies (aOR, 2.88; 95% CI: 1.77–4.67), spontaneous pregnancies (aOR, 2.94; 95% CI: 1.51–5.75), cohabitants drinking alcohol daily (aOR, 2.01; 95% CI: 1.09–3.70), and those using tobacco in the first trimester (aOR, 5.37; 95% CI: 2.70–10.66) were more likely to report alcohol use. In addition, women with a lower education (aOR, 7.67; 95% CI: 3.76–15.67), unplanned pregnancies (aOR, 3.31; 95% CI: 1.53–7.15), cohabitants using tobacco (aOR, 9.11; 95% CI: 4.48–18.52), and those who used alcohol (aOR, 6.67; 95% CI: 3.07–14.64) or illicit drugs (aOR, 39.03; 95% CI: 3.72–409.83) in the first trimester were more likely to report tobacco use. **Conclusions:** Despite a significant reduction in substance use in pregnancy compared to before pregnancy, a relevant portion of women continue to use substances, particularly in the early stages before pregnancy awareness. Targeted public health interventions and (more) awareness among caregivers are needed to further promote substance use cessation before conception.

## 1. Introduction

Substance use during pregnancy, including alcohol, tobacco and illicit drug use, is a significant public health concern due to its association with adverse maternal (miscarriage, preterm birth, or placental abruption) [1,2,3,4,5,6] and neonatal outcomes (small for gestational age (SGA), prematurity, or fetal alcohol syndrome (FAS)) [1,2,3,4,5,6,7,8]. Likewise, maternal complications such as maternal anemia and postpartum depression have been associated with perinatal substance use [4,6,9,10,11]. Substance use in pregnancy, even in small amounts, has also been linked to teratogenic effects, including craniofacial abnormalities, organ malformations and neurodevelopmental impairment such as lower IQ or behavioral problems [2,3,7,8,12]. Additionally, substance use prior to conception, which may be linked to poorer maternal preconception health, has been associated with adverse pregnancy and neonatal outcomes [13,14,15]. These findings emphasize the importance of avoiding substance use before and in pregnancy to ensure optimal maternal and infant health.

Although adverse effects following substance use during pregnancy have been extensively described, pregnant women may be somewhat unaware of the potential impact of the associated risks or will not value the risk as important. In 2016, only 48% of the pregnant women (in their 20th week of pregnancy) who received care in a public university hospital in Spain knew that the teratogenic effects of prenatal alcohol exposure are lifelong and 27.1% could not specify any risks when asked [16]. Furthermore, a low perception of risks was correlated with more frequent alcohol use [16]. As a safe lower limit for alcohol has not been defined, health authorities worldwide, including the World Health Organization [17], recommend complete abstinence from alcohol use during pregnancy. Similarly, abstinence from tobacco and illicit drug use is also strongly advised.

Despite the recommendations of avoiding any substance use during pregnancy, the most recent data from Belgium collected in 2016 showed that substance use in a highly educated cohort of pregnant women visiting a tertiary obstetrics clinic was prevalent, with about 6% reporting alcohol or tobacco use during the previous week (almost equally divided over the gestational trimesters) [18]. Given the outdated nature of these findings and the well-known adverse effects of substance use in pregnancy, updated and more comprehensive data were urgently needed to reflect current prevalences and identify determinants of such use in Belgium. In fact, insights into substance use in pregnancy are vital to assess the need for public health interventions, while identifying specific subgroups which should be primarily targeted.

Therefore, this study aimed to determine the current prevalence of alcohol, tobacco and illicit drug use before and during pregnancy in Belgium, as well as the characteristics associated with alcohol and tobacco use during pregnancy.

## 2. Materials and Methods

### 2.1. Study Design and Population

An observational study was performed using data collected in the BELpREG pregnancy registry (see www.belpreg.be/en, accessed on 3 December 2024). The BELpREG registry is the only ongoing, prospective, longitudinal, perinatal cohort in Belgium using digital self-reported questionnaires to collect real-world data on periconceptional exposures and mother–infant outcomes. BELpREG aims to gain more knowledge on the utilization and safety of exposure to medications, vaccines, health products and other substances around pregnancy [19]. BELpREG was established in November 2022. All pregnant persons 18 years or older and receiving healthcare in Belgium are considered eligible to participate in the BELpREG cohort. Upon enrolment, participants complete the first (“enrolment”) questionnaire, followed by subsequent (“follow-up”) questionnaires every four weeks during pregnancy until eight weeks postpartum. Additionally, participants receive two questionnaires on child health and development at 6 and 12 months after birth. The BELpREG questionnaires were developed using the core data elements compiled within the IMI ConcePTION project [20] and supplemented with variables defined by the European Medicines Agency and existing, similar registration systems [21]. Individuals can enroll in BELpREG at any time during pregnancy, although enrolment as early on as possible in pregnancy is preferred. BELpREG recruits pregnant women through various channels, including social media (advertising), healthcare professional referrals and via printed and digital posters and flyers. BELpREG questionnaires are available in Dutch, French and English (with the French and English translations added in January 2024). More details on the development and design of the BELpREG pregnancy registry have previously been described elsewhere [19,22].

Data extraction for this study took place on 13 August 2024. All BELpREG participants who had completed at least one questionnaire on substance use during pregnancy by the time of data extraction were included in this study. The number of questionnaires completed by each participant varied between one and eleven, as enrolment could occur at any time during pregnancy. Further, participants were not required to already have delivered. Participants who had not completed the substance use questions at enrolment, or missed a follow-up questionnaire in pregnancy, could still complete questions related to substance use in a later follow-up questionnaire and hence, were included in this study.

Ethics approval was obtained from EC Research UZ/KU Leuven (S66464); all participants provided an electronic informed consent prior to study enrolment. The study is reported using the STROBE checklist (see Supplementary Material S1) [23].

### 2.2. Study Outcomes

Participants were asked about their exposure to alcohol, tobacco (i.e., defined as cigarettes or vaping, the latter defined as e-cigarettes containing nicotine) and illicit drug use in the preconception period (i.e., defined as the year before conception), during the 1st trimester (i.e., gestational week 1–12), 2nd trimester (i.e., week 13–26) and 3rd trimester (i.e., week 27 until the last follow-up questionnaire before delivery).

*Preconception* use of alcohol, tobacco and illicit drug use was assessed at the enrolment questionnaire by asking questions on any exposures in the year before pregnancy (yes/no).

Alcohol, tobacco and illicit drug use *during* pregnancy were queried in the enrolment and four-weekly follow-up questionnaires in pregnancy (yes/no). Participants who reported not using any substances during pregnancy at the enrolment questionnaire were asked about the timing of alcohol, tobacco and illicit drug use cessation (i.e., in the year before pregnancy, since trying to get pregnant, since I knew I was pregnant). Additional questions focused on the amount of alcohol and tobacco use during pregnancy. More specifically, with respect to alcohol, the amount of alcohol use was asked based on the following three questions: (1) the frequency of drinking alcohol during pregnancy (i.e., monthly or less, 2–4 times a month, 2–3 times a week, 4 or more times a week), (2) the number of standard glasses of alcohol (i.e., defined as the amount of a drink that is usually served in a pub or at a restaurant, e.g., 25cl beer or 10cl wine) used per occasion (i.e., <1, 1 or 2, 3 or 4, 5 or 6, 7 or 9, 10 or more) and (3) binge drinking during pregnancy, which was defined as the consumption of six or more standard glasses of alcohol on one occasion (i.e., never, less than monthly, monthly, weekly, daily or almost daily). With respect to tobacco use, the amount of tobacco used was assessed based on the mean daily number of cigarettes between two consecutive BELpREG questionnaires. The categories 1–10 cigarettes/day, 11–20 cigarettes/day, 21–30 cigarettes/day, >30 cigarettes/day were used until July 2024, after which the following modified categories were used to obtain more detailed data: <1 cigarette/day, 1–5 cigarettes/day, 6–10 cigarettes/day, 11–20 cigarettes/day and >20 cigarettes/day. Lastly, the type of illicit drugs used in pregnancy was questioned (i.e., cannabis, XTC, amphetamines, hallucinogens, cocaine, ketamine, heroine, GHB, or other).

The questionnaires on substance use were based on established European standards (e.g., with respect to binge drinking), and were further refined with the input from expert centers in our country (i.e., Flemish Expertise Centre for Alcohol and Other Drugs and the Flemish Institute for Healthy Living) [19]. A detailed overview of the substance use-related questions is provided in Supplementary Material S2.

### 2.3. Covariates

The following covariates, for which an association with either alcohol or tobacco use during pregnancy has previously been reported, were available in and extracted from the enrolment questionnaire: maternal age [24,25,26,27,28,29,30,31,32,33,34], maternal ethnical background [26,28,31,32,34,35,36], marital status [29,30,37,38], maternal education [25,26,29,30,34,36,37,38,39,40,41], paternal education [31], maternal employment in the past year [30,31,40,41], household annual gross income [25,37], preconception body mass index (BMI) [26,28,36], chronic condition prior to pregnancy [26], (un)planned pregnancy [27,29,31], method of conception [28], gravidity [25,26,28,29,31,40], previous planned termination of pregnancy [32], intention to breastfeed [31] and substance use among cohabitants [31,41]. A detailed overview of the questions related to these covariates is provided in Supplementary Material S2.

### 2.4. Data Handling and Analysis

Descriptive statistics were used to determine the characteristics of the total study population and the participants who had reported any substance use in the first trimester.

Descriptive statistics were also used to report the prevalence of alcohol, tobacco and illicit drug use in the preconception period, in each trimester and during pregnancy. To calculate the timing of substance use during pregnancy, gestational age was defined based on the expected delivery date, which was determined by ultrasound (as the preferred option), the start date of the last menstrual period, or the moment of conception in case of assisted reproductive technology (ART), along with the date of survey completion. The prevalence for each trimester was calculated based on the gestational age at the moment of survey completion. The prevalence for each trimester was determined using data of the enrolment and/or follow-up questionnaires of participants who had fully completed that trimester and who had provided data on substance use during that specific trimester. For the calculation of the prevalence of the entire pregnancy, only participants who had given birth by the time of data extraction were considered by combining all registrations of substance use obtained during pregnancy. With respect to binge drinking, this variable was dichotomized (yes/no) and also used for the calculations per trimester or total pregnancy.

The amount of alcohol and tobacco use during pregnancy was analyzed as follows. First, the total number of standard glasses of alcohol was calculated considering the frequency of alcohol use and the number of glasses of alcohol used per occasion. The latter two questions were combined to the number of standard glasses of alcohol per day. Taking into account the number of days between two consecutive questionnaires, the total number of standard glasses over this period was calculated. Second, the mean number of cigarettes per day was calculated based on the mean daily number of cigarettes reported across the various questionnaires/periods. Therefore, the mean number of cigarettes per day was recoded from never used tobacco, <1, 1–5, 1–10, 6–10, 11–20, 21–30, >20 and >30 cigarettes per day into 0, 0.5, 2.5, 5, 7.5, 15, 25, 25 and 35 cigarettes per day choosing the midpoint, to be able to pool data from the initial and modified response categories [42,43]. The time period for alcohol and tobacco use was determined in the same way as for the calculation of the prevalence of alcohol, tobacco and illicit drug use.

In addition to descriptive statistics, backward multivariable logistic regressions were applied to determine characteristics associated with alcohol and tobacco use in the first pregnancy trimester. Only participants who reported any alcohol or tobacco use during pregnancy and who were beyond the first trimester by the time of data extraction were considered for the regression analysis (i.e., dependent variable). Regressions were only performed if numbers allowed, which was the case for alcohol and tobacco but not for illicit drug use. The following independent variables were used in the models: maternal age, maternal ethnical background, marital status, maternal education, preconception BMI, chronic condition before pregnancy, (un)planned pregnancy, method of conception, gravidity, previous planned termination of pregnancy, daily alcohol drinking cohabitant, a tobacco-using cohabitant, an illicit drug-using cohabitant, preconception alcohol use, preconception tobacco use, alcohol use during the 1st trimester, tobacco use during the 1st trimester and illicit drug use in the 1st trimester. Non-significant covariates (*p* > 0.05) were removed from the model one-by-one until only significant covariates remained. Collinearity among the covariates was checked using Pearson correlation coefficients, with a cut-off value of 0.90. The results are shown as adjusted odds ratios (aORs) with 95% confidence intervals (95% CI). A sensitivity analysis was conducted to identify determinants of alcohol and tobacco use in the first trimester, utilizing univariable and multivariable logistic regressions with a stepwise approach. Covariates with a *p*-value < 0.10 in the univariable analyses were included in the multivariable models. Statistical analyses were conducted using IBM SPSS Statistics version 29.

## 3. Results

### 3.1. Characteristics of the Study Population

**Figure 1:**
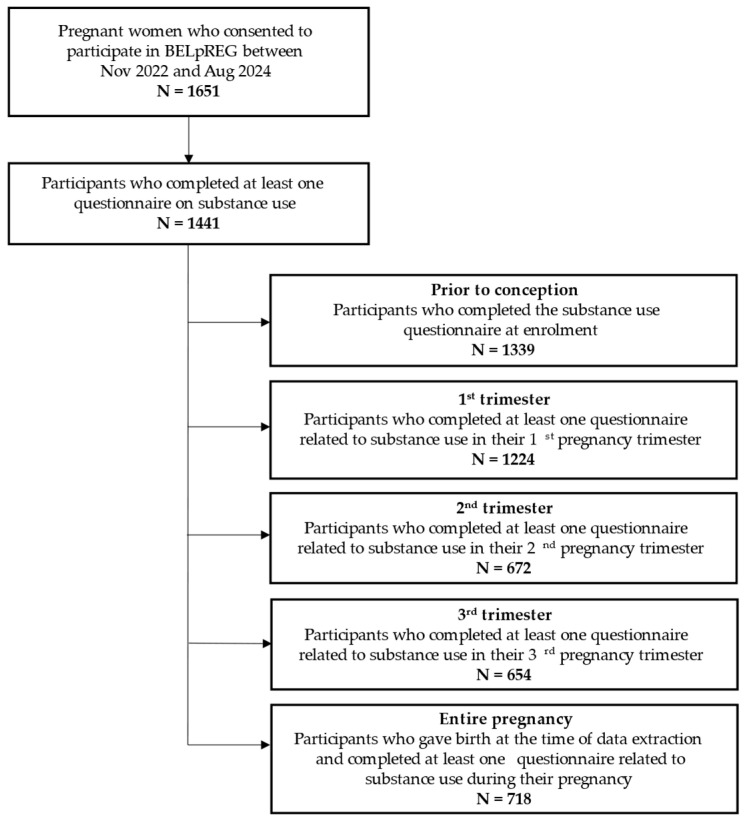
Overview of the study sample, highlighting the five different groups used for the calculation of the prevalence of substance use prior to and during pregnancy (i.e., 1st, 2nd and 3rd trimesters and entire pregnancy).

In total, 1651 pregnant women had been enrolled in the BELpREG cohort by 13 August 2024. Out of these, 1441 women had completed at least one questionnaire on substance use and were included for this study. Figure 1 provides an overview of the study sample, highlighting the number of participants used for the calculation of the prevalence of substance use prior to and during pregnancy (first, second, and third trimesters and entire pregnancy).

Table 1 presents the characteristics of the study population in general and according to alcohol and tobacco use in the first trimester. About half of the participants were aged between 30 and 34 years (50.9%). Most participants identified as Caucasian (96.9%) and had a partner (95.4%). Further, 85.2% had a high education level. Among participants with a partner, 68.3% of their partners were also highly educated. The employment rate in the past year among participants was 95.2%.

With respect to maternal health, 57.2% had a preconception BMI between 18.5 and 25 kg/m^2^. A chronic condition prior to pregnancy was reported by 38.2% of the participants. Among those participants suffering from chronic conditions, the most common conditions were allergic rhinitis (due to pollen) (15.5%), asthma (11.3%), migraine (11.1%), allergic or hypersensitivity conditions (8.0%) and hypothyroidism (7.1%).

Moreover, most of the pregnancies were planned (89.9%), 52.7% of the participants were multigravidae and 17.8% cited undergoing assisted reproductive technology (ART) prior to the index pregnancy. A previous planned termination of pregnancy was reported by 12.4% of multigravida participants. Lastly, the intention to breastfeed at the time of enrolment in the BELpREG registry was high (83.2%).

Upon enrolment in the BELpREG registry, 5.1% indicated having a cohabitant who was drinking alcohol daily (i.e., at the time the pregnant woman enrolled in the pregnancy registry), 12.0% reported having a cohabitant using tobacco and 2.4% answered having a cohabitant who was using illicit drugs. The median gestational age at enrolment in the BELpREG registry, at the time of data extraction, was 16 weeks (IQR (interquartile range): 10–25 weeks).

### 3.2. Prevalences of Alcohol, Tobacco and Illicit Drug Use

Table 2 shows the prevalence of alcohol, tobacco and illicit drug use before and during pregnancy. Regarding exposure *prior to conception*, 82.2% reported alcohol use, 10.0% tobacco use and 4.2% illicit drug use.

*During the first trimester*, the self-reported prevalence of alcohol, tobacco and illicit drug use was 12.9%, 4.1% and 0.6%, respectively. *During the second trimester*, alcohol use was reported by 3.6%, tobacco use by 2.0% and illicit drug use by 0.6%. Prevalences for the *third trimester* were 4.7% for alcohol use, 2.1% for tobacco use and 0.2% for illicit drug use. Considering the entire pregnancy (i.e., including only women who had already delivered by the time of data extraction), 14.6% of the participants reported alcohol use, 4.0% tobacco use and 0.4% illicit drug use during pregnancy. Notably, pregnant women who reported substance use cessation only after becoming aware of their pregnancy were excluded from these prevalence calculations.

Further, binge drinking was reported by 10.0% of the women prior to conception, 4.1% in the first trimester, 2.0% in the second trimester, 2.1% in the third trimester and 4.6% over the entire pregnancy.

Finally, the few women who reported the use of illicit drugs during pregnancy (n = 7) had used cannabis (n = 5), cocaine (n = 3) and ketamine (n = 1). Among these women, two used more than one illicit drug during pregnancy.

### 3.3. Quantity of Alcohol and Tobacco Use During Pregnancy

Table 2 illustrates the amount of self-reported alcohol and tobacco used in pregnancy. Nearly half of the participants who had delivered by the time of data extraction (48.6%) reported having drunk 1–5 standard glasses of alcohol *throughout the entire pregnancy*, 21.0% less than 1 glass and 16.2% 5–10 glasses. With regard to the first trimester, 25.3% consumed less than 1 glass, 48.1% 1–5 glasses and about 1/4 more than 5 standard glasses.

For tobacco use, the mean number of cigarettes used per day *in the first trimester* for most women using tobacco (90.0%) was 1–5 cigarettes. Similar results were found for the second and third trimester. With regard to the entire pregnancy, about half of the women using tobacco consumed on average less than 1 cigarette a day or 1–5 cigarettes a day. However, when the maximum reported mean number of cigarettes per day was considered, much higher amounts were observed. More specifically, some women reported having used up to 10–20 cigarettes per day, corresponding to 14.0% in the first trimester, 16.7% in the second trimester, 21.4% in the third trimester and 20.7% over the entire pregnancy.

### 3.4. Prevalences of Early Pregnancy Exposures Before Pregnancy Awareness

Participants who reported substance use prior to but not in pregnancy were asked when they stopped using the substance(s). Overall, 32.3% (n = 344) of participants who indicated that they had not consumed alcohol during pregnancy reported that they ceased alcohol use only after being aware of their pregnancy. Adding this early-pregnancy exposure to the reported exposures during pregnancy (as detailed in Table 2), the total prevalence of any alcohol use in the first trimester was 41.0% (n = 344). Similarly, for tobacco and illicit drug use, 2.6% (n = 31) and 0.7% (n = 8) of participants, respectively, who reported no use in pregnancy admitted to quitting only after becoming aware of their pregnancy. This led to a first-trimester prevalence of any tobacco or illicit drug use of 6.6% (n = 81) and 1.2% (n = 15), respectively. In this cohort, the median gestational age at the time of pregnancy awareness was 4 weeks (IQR: 4–5 weeks).

### 3.5. Determinants of Alcohol Use in the First Pregnancy Trimester

Table 3 presents the associations between maternal characteristics and alcohol use in the first trimester. Tobacco use in the first trimester, (un)planned pregnancy, daily alcohol use by cohabitant(s), maternal education and the method of conception were found to be associated with alcohol use in the first trimester.

An increased likelihood of alcohol use in the first trimester was found for the following covariates: tobacco use in the first trimester, spontaneous pregnancy, unplanned pregnancy, high education level and alcohol use by cohabitant(s). First, tobacco use in the first trimester strongly increased the odds of alcohol use in the first trimester (aOR, 5.37; 95% CI: 2.70–10.66). Second, women who conceived spontaneously were more likely to drink alcohol in the first trimester (aOR, 2.94; 95% CI: 1.51–5.75). Further, an unplanned pregnancy was associated with a nearly threefold increase in the likelihood of alcohol use (aOR, 2.88; 95% CI: 1.77–4.67), while pregnant participants with a high level of education were also more likely to drink alcohol in the first trimester (aOR, 2.12; 95% CI: 1.14–3.96). Finally, having a cohabitant who was drinking alcohol daily doubled the odds of alcohol use by the pregnant woman herself (aOR, 2.01; 95% CI: 1.09–3.70).

All participants who had reported alcohol use in the first trimester also cited alcohol use in the year prior to conception. Hence, preconception alcohol use is an important determinant but could not be included in the regression analysis due to statistical limitations.

### 3.6. Determinants of Tobacco Use in the First Pregnancy Trimester

Table 4 presents the associations between maternal characteristics and tobacco use in the first trimester. Tobacco use by a cohabitant, alcohol use in the first trimester, maternal education, (un)planned pregnancy and illicit drug use in the first trimester were found to be associated with tobacco use in the first trimester.

First, a tobacco-using cohabitant strongly increased the odds of tobacco use in the first trimester (aOR, 9.11; 95% CI: 4.48–18.52). Second, alcohol use in the first trimester was strongly associated with tobacco use in the same period (aOR, 6.67; 95% CI: 3.07–14.64). Third, pregnant participants with a low or medium level of education were more likely to use tobacco in the first trimester (aOR, 7.67; 95% CI: 3.76–15.67). Moreover, an unplanned pregnancy was associated with a threefold increase in the likelihood of tobacco use in the first trimester (aOR, 3.31; 95% CI: 1.53–7.15). Finally, although the number of illicit drug users in the first trimester was very limited in our cohort, significantly increased odds of tobacco use in the first trimester were observed among such drug users, despite a very wide confidence interval (aOR, 39.03; 95% CI: 3.72–409.83).

Similar to alcohol use in the first trimester, all participants who reported tobacco use in the first trimester also indicated tobacco use in the year prior to conception. As a result, preconception tobacco use is an important determinant but could not be included in the regressions due to statistical limitations.

A sensitivity analysis was conducted to identify determinants of alcohol and tobacco use in the first trimester using multivariable logistic regressions with a *stepwise approach*. This stepwise method identified the same determinants as presented for both alcohol use (Table S3 in the Supplementary Material S3) and tobacco use during the first trimester (Table S4 in the Supplementary Material S3), except for maternal education level, which was no longer observed as a determinant for alcohol use during the first trimester.

## 4. Discussion

### 4.1. Main Findings

This study aimed to provide evidence on the prevalence of alcohol, tobacco and illicit drug use before and during pregnancy in Belgium and determinants of alcohol and tobacco use during pregnancy. The data used in this study had recently been collected by the BELpREG pregnancy registry in Belgium and therefore provide an up-to-date and comprehensive view on this relevant topic in a Western European country.

First, the study found that 82.2% of the pregnant women reported alcohol use, 10.0% tobacco use and 4.2% illicit drug use in the year prior to conception. In our study, alcohol use in the year before pregnancy (82.2%) was somewhat higher compared to the general population of women in Belgium (70.1%) [44], illustrating that alcohol use seems to be the norm in Belgium and is socially accepted. Tobacco use rates (10.0%), in the year prior to conception, were slightly lower compared to the general population (14.6%) [45].

In the first trimester, the self-reported prevalence of alcohol, tobacco and illicit drug use dropped to 12.9%, 4.1% and 0.6%, respectively. Overall, the prevalence of alcohol use was in line with previous studies [18,40,46,47]. Similar prevalences on binge drinking, as observed in our study, have also previously been reported [46]. Moreover, tobacco use in pregnancy was found to be on the lower side of the range of previously found prevalence estimates [28,29,31,32,33,34,38,41,48]. In contrast, illicit drug use in pregnancy was found to be ten times lower in our study cohort compared to previous studies from other countries [47,49]. The trend of a lower exposure to illicit drugs was, in our cohort, also reflected by the numbers prior to conception and might be explained by the highly educated sample and different drug regulations across countries affecting the (reporting of) use [47,49,50].

Nevertheless, it is clear that a significant proportion of pregnant women continue to use substances until pregnancy identification. Specifically, 32.3% of women ceased alcohol use only after learning about their pregnancy in the first trimester. The prevalences for tobacco use (2.6%) and use of illicit drugs (0.7%) until pregnancy awareness were much lower. This resulted in a composite, total prevalence of alcohol use in the first trimester of 41.0%, for tobacco use of 6.6% and for illicit drug use of 1.2%. Although previous studies reported even higher prevalences of alcohol use in pregnancy before pregnancy awareness [27,51], our observed numbers are alarming as they show that early pregnancy exposures to substances in the first and most vulnerable weeks of pregnancy still very often occur in our pregnant population. This underscores the need for more emphasis and dissemination to the public on the importance of preconception care, including the recommendations of total abstinence from alcohol, tobacco and illicit drug use [27,51].

When looking at the quantities of alcohol and tobacco use in pregnancy, however, the used amount was generally rather limited. About 21.0% cited drinking less than one standard glass of alcohol in the entire pregnancy and about half of the participants reported drinking one to five standard glasses in total. Some pregnant women described alcohol use as “having a sip of an alcoholic beverage” or “accidently receiving an alcoholic drink instead of a non-alcoholic version.” Similarly, 44.8% of participants who reported tobacco use indicated an average consumption of less than one cigarette per day throughout their pregnancy. In contrast, 51.7% of smokers indicated an average use of one to five cigarettes per day over the course of their pregnancy.

With respect to potential determinants, women with a high education level, an unplanned pregnancy, a spontaneous pregnancy, a daily alcohol drinking cohabitant and tobacco use in the first trimester were more likely to drink alcohol in the first trimester. Similar findings were previously reported for education level [37,39,40], pregnancy planning [27] and tobacco use in pregnancy [24,26,35]. Similar trends have also previously been observed in the general population in Belgium and elsewhere, where individuals with higher education levels tend to have higher alcohol consumption rates [44,52]. This has been argued to be a result of greater gender equality in highly developed countries [52]. Further, women with a low/medium education level, an unplanned pregnancy, a tobacco-using cohabitant and using alcohol or illicit drugs in the first trimester were more likely to use tobacco in the first trimester. Many similarities can be drawn with previous studies showing that women with low/medium levels of education [29,30,31,32,34,38,41], those who had an unplanned pregnancy [29,31], those with a tobacco-using cohabitant [41] and those using alcohol or illicit drugs in the first trimester [29,31] were more likely to use tobacco in the first trimester. Finally, all participants with self-reported alcohol or tobacco use in the first trimester already used these substances in the year prior to conception.

### 4.2. Methodological Considerations

This study has some strengths. First, we used a large, longitudinal cohort, enhancing the trustworthiness of the findings. Second, the use of anonymous data reported to the BELpREG registry likely reduced information or social desirability bias, leading to more accurate and reliable data. Moreover, BELpREG questionnaires are sent out every four weeks in pregnancy, minimizing the risk of recall bias. Participants are also required to complete the substance use-related questions in the BELpREG questionnaires, limiting the possibility of missing values. Next, when designing the BELpREG questionnaires related to alcohol, tobacco and illicit drug use, input was sought from the respective centers of expertise in our country on these matters (i.e., Flemish Expertise Centre for Alcohol and Other Drugs and the Flemish Institute for Healthy Living), with the aim of collecting comprehensive and reliable self-reported data on substance use [19]. Further, our study not only provides up-to-date evidence on the extent of the use of various substances during the entire pregnancy but also about substance use before pregnancy awareness, which may not be perceived by women as substance use in pregnancy or after conception. Lastly, a sensitivity analysis was conducted using a different statistical approach for the multivariable logistic regressions, enhancing the robustness and reliability of the findings.

Some limitations should be addressed. First, self-reported data may entail the risk of reporting bias. Currently, no objective measurements or comparisons are in place to verify the self-reported data on substance use in BELpREG. Second, selection bias occurred given the higher education level and employment rates in our sample, limiting the generalizability of the findings. When interpreting the findings from the BELpREG cohort, demographics of participants should be considered related to population statistics. The BELpREG cohort has a higher proportion of mothers aged 30–34 years, while mothers aged 18–24 years are less represented [53]. The BELpREG cohort also contains more highly educated individuals with higher household income levels, has higher employment rates and more women with a Caucasian ethnicity [54,55,56]. Finally, in BELpREG, 17.8% of the pregnancies did not occur spontaneously, which is nearly double the population statistic of 9.6% [53]. It cannot be excluded that these differences affected the prevalence of substance use in pregnancy. For example, while the overrepresentation of highly educated women may have led to an overestimation of the prevalence of alcohol consumption, the underrepresentation of both spontaneous and unplanned pregnancies and of women with lower education levels may have led to an underestimation of the actual numbers of alcohol consumption and smoking in the perinatal population, respectively. In addition, the BELpREG registry only became accessible to French-speaking individuals from January 2024 onwards, resulting in an underrepresentation of French-speaking individuals (10.6%) and challenging the generalizability of the results for the entire country. For instance, regarding the general population in Belgium, alcohol use is higher in Flanders, while smoking rates are higher in Wallonia [44,45]. Due to the low number of illicit drug users, an exploration of determinants was not possible. Additionally, individual substance use trajectories in pregnancy were not studied. Given the overall low numbers of substance-exposed women in pregnancy, especially after pregnancy awareness and throughout pregnancy, the interpretation of individual trajectories in this cohort might be, for the time being, challenging. A backward regression approach also comes with some limitations, such as the risk of overfitting, the loss of important variables and ignoring interaction terms and the likelihood that variables with a strong univariate link are predominantly retained. Moreover, no questions were included on the amount of vaping and illicit drug use. Data on any substance use between the last questionnaire in pregnancy and delivery were also lacking. Consequently, prevalence estimates of substance use in the third trimester and over the entire pregnancy might be an underestimation. Yet, this is considered rather unlikely, as de novo substance use initiated in the weeks between the last questionnaire in pregnancy and the delivery is improbable. Lastly, no data were available on the amount of alcohol or tobacco use in pregnancy among women who reported substance use cessation since pregnancy awareness, so no conclusions can be drawn about the extent of alcohol and tobacco use by these women in early pregnancy, requiring further investigation.

Interestingly, Table S5 (see Supplementary Material S4) provides a comparison of the characteristics between participants who had delivered and those who had not yet delivered or were lost to follow-up by the time of data extraction. Overall, no significant differences were observed between the two groups. Given the lack of differences and the larger sample size of first-trimester pregnant women, the latter group was chosen to find determinants associated with substance use, thereby improving statistical power and precision. Moreover, most exposures to substances occurred in the first trimester and data completeness was higher for this period compared to the entire pregnancy.

Finally, the relationship between substance use before or in pregnancy and the occurrence of adverse pregnancy or neonatal outcomes was beyond the scope of this study.

### 4.3. Future Perspectives

The findings underscore the need for targeted interventions to improve public health. All pregnant women who reported alcohol or tobacco use in the first trimester also indicated alcohol or tobacco use in the year prior to conception. Additionally, most exposures to substances occurred prior to conception or in early pregnancy before pregnancy awareness. These findings underscore the need for robust preconception interventions aimed at promoting healthy lifestyle (changes) before conception [57,58]. Interestingly, unplanned pregnancy was identified as a determinant of both alcohol and tobacco use in pregnancy, prompting the need to optimize the use of effective (hormonal) contraception in the population and to consider interventions to prevent unplanned pregnancies. Future research should explore which tailored interventions can be designed to address pregnancy planning and preconception health and to minimize unplanned pregnancies [57,58].

As polysubstance use in the first trimester occurred, interventions should specifically target pregnant women using various substances simultaneously. Early identification and support for these women can help mitigate the risks. Tailored programs addressing the specific needs and challenges faced by these individuals could be more effective in promoting positive lifestyle changes [59]. Interventions should also focus on the social environment of pregnant women, as alcohol or tobacco use by cohabitants relates to maternal tobacco and alcohol use in pregnancy. Therefore, multicomponent family-based interventions are needed. Recently, the Born in Belgium Professionals tool was designed to help healthcare professionals in Belgium detect antenatal psychosocial vulnerabilities during pregnancy [60,61]. This tool also includes a comprehensive screening for substance use and has the potential to direct pregnant women to personalized professional care.

In future studies, when our BELpREG cohort has grown further, we aim to include a broad range of mental health disorders and related covariates for regression analyses, as these conditions might influence substance use. Likewise, individual substance use trajectories could also be further studied.

## 5. Conclusions

This study highlighted the prevalence and determinants of alcohol, tobacco and illicit drug use before and during pregnancy in Belgium. Despite a significant reduction in substance use in pregnancy, a notable portion of women continue to use substances until they become aware of their pregnancy. Determinants associated with substance use in the first gestational trimester include substance use by cohabitants, maternal education level, (un)planned pregnancies and method of conception. The results underscore the need for policy makers to enable targeted public health interventions to further enhance substance use cessation before conception. Healthcare providers should be aware of these findings and proactively address substance use during counseling sessions, both before and in the early stages of pregnancy.

## Figures and Tables

**Table 1:** Characteristics of the study population in general and after stratification according to self-reported alcohol and tobacco use in the first pregnancy trimester.

Characteristics	All Pregnant Women (N = 1441)		Pregnant Women Who Used Alcohol in the 1st Trimester ^1^ (N = 158)		Pregnant Women Who Used Tobacco in the 1st Trimester ^1^ (N = 50)	
	N	%	N	%	N	%
**Sociodemographic variables**						
Maternal age (in years)						
18–24	27	1.9	4	2.5	3	6.0
25–29	449	31.2	50	31.6	15	36.0
30–34	733	50.9	82	51.9	20	40.0
35–39	176	12.2	21	13.3	11	22.0
≥40	21	1.5	1	0.6	1	2.0
Missing	35	2.4	-	-	-	-
Maternal ethnical background						
Caucasian	1397	96.9	157	99.4	48	96.0
Non-Caucasian or I do not know	19	1.4	1	0.6	2	4.0
Missing	25	1.7	-	-	-	-
Marital status						
Partner	1375	95.4	157	99.4	47	94.0
No partner	41	2.8	1	0.6	3	6.0
Missing	25	1.7	-	-	-	-
Maternal education ^2^						
Low/medium	188	13.1	20	12.7	29	58.0
High	1228	85.2	138	87.3	21	42.0
Missing	25	1.7	-	-	-	-
Paternal education ^2^						
Low/medium	429	31.2	41	26.1	30	65.2
High	939	68.3	116	73.9	16	34.8
Missing	7	0.5	-	-	-	-
Maternal employment status in the past year						
Employed	1372	95.2	156	98.7	50	100.0
Not employed	44	3.1	2	1.3	0	0.0
Missing	25	1.7	-	-	-	-
Annual household gross income						
EUR < 45,000	171	11.9	19	12.0	11	22.0
EUR 45,000–65,000	342	23.7	30	19.0	17	34.0
EUR > 65,000	562	39.0	76	48.1	7	14.0
I do not know/I would rather not tell	341	23.7	33	20.9	15	30.0
Missing	25	1.7	-	-	-	-
Maternal preconception BMI (kg/m^2^)						
<18.5	48	3.3	4	2.5	1	2.0
18.5–25	824	57.2	101	63.9	27	54.0
25–30	342	23.7	37	23.4	14	28.0
>30	201	13.9	16	10.1	8	16.0
Missing	26	1.8	-	-	-	-
Chronic medical condition prior to pregnancy ^3^						
Yes	550	38.2	51	32.3	20	40.0
No	866	60.1	107	67.7	30	60.0
Missing	25	1.7	-	-	-	-
**Pregnancy-related variables**						
Planned pregnancy						
Yes	1295	89.9	125	79.1	33	66.0
No	121	8.4	25	20.9	17	34.0
Missing	25	1.7	-	-	-	-
Method of conception						
Spontaneous	1159	80.4	148	93.7	47	94.0
ART ^4^	257	17.8	10	6.3	3	6.0
Missing	25	1.7	-	-	-	-
Gravidity						
Primigravida	657	45.6	76	48.1	18	36.0
Multigravida	759	52.7	82	51.9	32	64.0
Missing	25	1.7	-	-	-	-
Previous planned termination of pregnancy						
Yes	94	12.4	13	15.9	9	28.1
No	663	87.4	69	84.1	23	71.9
Missing	2	0.2	-	-	-	-
Intention to breastfeed						
Yes	1199	83.2	128	81.0	41	82.0
No or I do not know yet	217	15.1	30	19.0	9	18.0
Missing	25	1.7	-	-	-	-
**Environmental variables at the time of enrolment**						
Cohabitant who was drinking alcohol daily						
Yes	74	5.1	16	10.1	6	12.0
No	1265	87.8	142	89.9	44	88.0
Missing	102	7.1	-	-	-	-
Cohabitant who was using tobacco						
Yes	173	12.0	22	13.9	30	60.0
No	1166	80.9	136	86.1	20	40.0
Missing	102	7.1	-	-	-	-
Cohabitant who was using illicit drugs						
Yes	34	2.4	6	3.8	5	10.0
No	1305	90.6	152	96.2	45	90.0
Missing	102	7.1	-	-	-	-

^1^ Considering pregnant women who reported alcohol and/or tobacco use during the first trimester. ^2^ High education is defined as any formal degree obtained in higher education or university. ^3^ A chronic condition prior to pregnancy was defined as any diagnosed chronic condition before the start of the index pregnancy. ^4^ ART is defined as assisted reproductive technology.

**Table 2:** Prevalences and the amount of self-reported alcohol, tobacco and illicit drug use before and during pregnancy.

	Preconception ^1^ N = 1339	Trimester 1 ^2^ N = 1224		Trimester 2 ^2^ N = 972	Trimester 3 ^3^ N = 654	Total Pregnancy ^4^ N = 718
**Alcohol use**						
Prevalence	1100 (82.2%)	158 (12.9%)		35 (3.6%)	31 (4.7%)	105 (14.6%)
Total number of standard glasses						
<1		40 (25.3%)		2 (5.7%)	5 (16.1%)	22 (21.0%)
1–5		76 (48.1%)		16 (45.7%)	11 (35.5%)	51 (48.6%)
5–10		18 (11.4%)		11 (31.4%)	12 (38.7%)	17 (16.2%)
10–20		10 (6.3%)		1 (2.9%)	1 (3.2%)	9 (8.6%)
20–40		10 (6.3%)		5 (14.3%)	2 (6.5%)	4 (3.8%)
>40		4 (2.5%)		0 (0.0%)	0 (0.0%)	2 (1.9%)
Binge drinking ^5^		50 (4.1%)		7 (0.7%)	5 (0.8%)	33 (4.6%)
**Tobacco use**						
Prevalence	134 (10.0%)	50 (4.1%)		19 (2.0%)	14 (2.1%)	29 (4.0%)
Mean number of cigarettes per day						
<1		0 (0.0%)		1 (5.3%)	1 (7.1%)	13 (44.8%)
1–5		45 (90.0%)		17 (89.5%)	11 (78.6%)	15 (51.7%)
5–10		5 (10.0%)		1 (5.3%)	2 (14.3%)	1 (3.4%)
**Illicit drug use**						
Prevalence	56 (4.2%)	7 (0.6%)		6 (0.6%)	1 (0.2%)	3 (0.4%)

^1^ Preconception was defined as the year prior to conception; for this variable, all women who completed the enroll questionnaire on substance use were considered. ^2^ Per trimester: considering all women who were beyond the first or second trimester at the time of data extraction and provided data on substance use during the respective trimester. ^3^ Third trimester: considering all women who had already given birth at the time of data extraction and provided data on substance use during the third trimester. ^4^ Total pregnancy: considering all women who had already given birth at the time of data extraction. ^5^ Binge drinking was defined as the consumption of ≥6 standard glasses of alcohol on one occasion.

**Table 3:** Associations between maternal characteristics and alcohol use during the first trimester.

	Alcohol Use in the 1st Trimester	
	aOR ^1^	95% CI
**Tobacco use in 1st trimester**		
Yes	5.37	2.70–10.66
**Method of conception**		
Spontaneous	2.94	1.51–5.75
**Planned pregnancy**		
No	2.88	1.77–4.67
**Maternal education**		
High	2.12	1.14–3.96
**Cohabitant who was drinking alcohol daily**		
Yes	2.01	1.09–3.70

^1^ aOR = adjusted odds ratios, adjusted for maternal education, planned pregnancy, method of conception, cohabitant who was drinking alcohol daily, or tobacco use during the 1st trimester. The results were derived by conducting a backward multivariable logistic regression where non-significant covariates (*p* > 0.05) were removed from the model one-by-one.

**Table 4:** Associations between maternal characteristics and tobacco use during the first trimester.

	Tobacco Use During the 1st Trimester	
	aOR ^1^	95% CI
**Cohabitant who was a tobacco user**		
Yes	9.11	4.48–18.52
**Maternal education**		
Low/medium	7.67	3.76–15.67
**Alcohol use in 1st trimester**		
Yes	6.67	3.07–14.64
**Planned pregnancy**		
No	3.31	1.53–7.15
**Illicit drug use in 1st trimester**		
Yes	39.03	3.72–409.83

^1^ aOR = adjusted odds ratios, adjusted for maternal education, (un)planned pregnancy, cohabitant who was a tobacco user, alcohol use in the 1st trimester, or illicit drug use in the 1st trimester. The results were derived by conducting a backward multivariable logistic regression where non-significant covariates (*p* > 0.05) were removed from the model one-by-one.

## Data Availability

The data presented in this manuscript are available on request from the corresponding author due to ethical and privacy reasons.

## Supplementary Materials

The following supporting information can be downloaded at: https://www.mdpi.com/article/10.3390/jcm14020613/s1, Table S1: STROBE checklist; Table S2: Overview questions; Tables S3 and S4: Sensitivity analysis; Table S5: Sample comparison.

## References

[1] Avsar T.S., McLeod H., Jackson L. (2021). Health outcomes of smoking during pregnancy and the postpartum period: An umbrella review. BMC Pregnancy Childbirth.

[2] Mamluk L., Edwards H.B., Savovic J., Leach V., Jones T., Moore T.H.M., Ijaz S., Lewis S.J., Donovan J.L., Lawlor D. (2017). Low alcohol consumption and pregnancy and childhood outcomes: Time to change guidelines indicating apparently ’safe’ levels of alcohol during pregnancy? A systematic review and meta-analyses. BMJ Open.

[3] Volkow N.D., Compton W.M., Wargo E.M. (2017). The Risks of Marijuana Use During Pregnancy. JAMA.

[4] Corsi D.J., Walsh L., Weiss D., Hsu H., El-Chaar D., Hawken S., Fell D.B., Walker M. (2019). Association Between Self-reported Prenatal Cannabis Use and Maternal, Perinatal, and Neonatal Outcomes. JAMA.

[5] Gabrhelik R., Mahic M., Lund I.O., Bramness J., Selmer R., Skovlund E., Handal M., Skurtveit S. (2021). Cannabis Use during Pregnancy and Risk of Adverse Birth Outcomes: A Longitudinal Cohort Study. Eur. Addict. Res..

[6] Gunn J.K., Rosales C.B., Center K.E., Nunez A., Gibson S.J., Christ C., Ehiri J.E. (2016). Prenatal exposure to cannabis and maternal and child health outcomes: A systematic review and meta-analysis. BMJ Open.

[7] O’Leary C.M. (2004). Fetal alcohol syndrome: Diagnosis, epidemiology, and developmental outcomes. J. Paediatr. Child Health.

[8] Feldman H.S., Jones K.L., Lindsay S., Slymen D., Klonoff-Cohen H., Kao K., Rao S., Chambers C. (2012). Prenatal alcohol exposure patterns and alcohol-related birth defects and growth deficiencies: A prospective study. Alcohol. Clin. Exp. Res..

[9] Pacho M., Aymerich C., Pedruzo B., Salazar de Pablo G., Sesma E., Bordenave M., Dieguez R., Lopez-Zorroza I., Herrero J., Laborda M. (2023). Substance use during pregnancy and risk of postpartum depression: A systematic review and meta-analysis. Front. Psychiatry.

[10] Pentecost R., Latendresse G., Smid M. (2021). Scoping Review of the Associations Between Perinatal Substance Use and Perinatal Depression and Anxiety. J. Obs. Gynecol. Neonatal. Nurs..

[11] Grzywacz E., Brzuchalski B., Smiarowska M., Malinowski D., Machoy-Mokrzynska A., Bialecka M.A. (2023). Significance of Selected Environmental and Biological Factors on the Risk of FASD in Women Who Drink Alcohol during Pregnancy. J. Clin. Med..

[12] Muggli E., Matthews H., Penington A., Claes P., O’Leary C., Forster D., Donath S., Anderson P.J., Lewis S., Nagle C. (2017). Association Between Prenatal Alcohol Exposure and Craniofacial Shape of Children at 12 Months of Age. JAMA Pediatr..

[13] Pielage M., El Marroun H., Odendaal H.J., Willemsen S.P., Hillegers M.H.J., Steegers E.A.P., Rousian M. (2023). Alcohol exposure before and during pregnancy is associated with reduced fetal growth: The Safe Passage Study. BMC Med..

[14] Lassi Z.S., Imam A.M., Dean S.V., Bhutta Z.A. (2014). Preconception care: Caffeine, smoking, alcohol, drugs and other environmental chemical/radiation exposure. Reprod Health.

[15] Zhou Q., Song L., Chen J., Wang Q., Shen H., Zhang S., Li X. (2021). Association of Preconception Paternal Alcohol Consumption With Increased Fetal Birth Defect Risk. JAMA Pediatr..

[16] Corrales-Gutierrez I., Mendoza R., Gomez-Baya D., Leon-Larios F. (2019). Pregnant Women’s Risk Perception of the Teratogenic Effects of Alcohol Consumption in Pregnancy. J. Clin. Med..

[17] World Health Organization (2014). Guidelines for the Identification and Management of Substance Use Disorders in Pregnancy.

[18] Ceulemans M., Van Calsteren K., Allegaert K., Foulon V. (2019). Health products’ and substance use among pregnant women visiting a tertiary hospital in Belgium: A cross-sectional study. Pharmacoepidemiol. Drug Saf..

[19] Sillis L., Foulon V., Allegaert K., Bogaerts A., De Vos M., Hompes T., Smits A., Van Calsteren K., Verbakel J.Y., Ceulemans M. (2023). Development and design of the BELpREG registration system for the collection of real-world data on medication use in pregnancy and mother-infant outcomes. Front. Drug. Saf. Regul..

[20] Richardson J.L., Moore A., Bromley R.L., Stellfeld M., Geissbuhler Y., Bluett-Duncan M., Winterfeld U., Favre G., Alexe A., Oliver A.M. (2023). Core Data Elements for Pregnancy Pharmacovigilance Studies Using Primary Source Data Collection Methods: Recommendations from the IMI ConcePTION Project. Drug. Saf..

[21] European Medicines Agency (2019). Guideline on Good Pharmacovigilance Practices (GVP) Product- or Population-Specific Considerations III: Pregnant and Breastfeeding Women.

[22] Ceulemans M., Sillis L., Allegaert K., Bogaerts A., De Vos M., Hompes T., Smits A., Van Calsteren K., Verbakel J.Y., Foulon V. (2024). Letter to the Editor re Davis et al., 2023: BELpREG, the first of its kind real-world data source on medication use in pregnancy in Belgium. Pharmacoepidemiol. Drug Saf..

[23] von Elm E., Altman D.G., Egger M., Pocock S.J., Gotzsche P.C., Vandenbroucke J.P., Initiative S. (2008). The Strengthening the Reporting of Observational Studies in Epidemiology (STROBE) statement: Guidelines for reporting observational studies. J. Clin. Epidemiol..

[24] Skagerstrom J., Alehagen S., Haggstrom-Nordin E., Arestedt K., Nilsen P. (2013). Prevalence of alcohol use before and during pregnancy and predictors of drinking during pregnancy: A cross sectional study in Sweden. BMC Public Health.

[25] Skagerstrom J., Chang G., Nilsen P. (2011). Predictors of drinking during pregnancy: A systematic review. J. Womens Health (Larchmt).

[26] Kitsantas P., Gaffney K.F., Wu H., Kastello J.C. (2014). Determinants of alcohol cessation, reduction and no reduction during pregnancy. Arch. Gynecol. Obs..

[27] McCormack C., Hutchinson D., Burns L., Wilson J., Elliott E., Allsop S., Najman J., Jacobs S., Rossen L., Olsson C. (2017). Prenatal Alcohol Consumption Between Conception and Recognition of Pregnancy. Alcohol. Clin. Exp. Res..

[28] Tsakiridis I., Mamopoulos A., Papazisis G., Petousis S., Liozidou A., Athanasiadis A., Dagklis T. (2018). Prevalence of smoking during pregnancy and associated risk factors: A cross-sectional study in Northern Greece. Eur. J. Public Health.

[29] Smedberg J., Lupattelli A., Mardby A.C., Nordeng H. (2014). Characteristics of women who continue smoking during pregnancy: A cross-sectional study of pregnant women and new mothers in 15 European countries. BMC Pregnancy Childbirth.

[30] de Wolff M.G., Backhausen M.G., Iversen M.L., Bendix J.M., Rom A.L., Hegaard H.K. (2019). Prevalence and predictors of maternal smoking prior to and during pregnancy in a regional Danish population: A cross-sectional study. Reprod Health.

[31] Riaz M., Lewis S., Naughton F., Ussher M. (2018). Predictors of smoking cessation during pregnancy: A systematic review and meta-analysis. Addiction.

[32] Sequi-Canet J.M., Sequi-Sabater J.M., Marco-Sabater A., Corpas-Burgos F., Collar Del Castillo J.I., Orta-Sibu N. (2022). Maternal factors associated with smoking during gestation and consequences in newborns: Results of an 18-year study. J. Clin. Transl. Res..

[33] Rumrich I.K., Vahakangas K., Viluksela M., Gissler M., Surcel H.M., Korhonen A., De Ruyter H., Hanninen O. (2019). Smoking during pregnancy in Finland—Trends in the MATEX cohort. Scand. J. Public Health.

[34] Kondracki A.J. (2019). Prevalence and patterns of cigarette smoking before and during early and late pregnancy according to maternal characteristics: The first national data based on the 2003 birth certificate revision, United States, 2016. Reprod Health.

[35] O’Keeffe L.M., Kearney P.M., McCarthy F.P., Khashan A.S., Greene R.A., North R.A., Poston L., McCowan L.M., Baker P.N., Dekker G.A. (2015). Prevalence and predictors of alcohol use during pregnancy: Findings from international multicentre cohort studies. BMJ Open.

[36] Kitsantas P., Gaffney K.F., Wu H. (2015). Identifying high-risk subgroups for alcohol consumption among younger and older pregnant women. J. Perinat. Med..

[37] Shmulewitz D., Hasin D.S. (2019). Risk factors for alcohol use among pregnant women, ages 15-44, in the United States, 2002 to 2017. Prev. Med..

[38] Houston-Ludlam A.N., Bucholz K.K., Grant J.D., Waldron M., Madden P.A.F., Heath A.C. (2019). The interaction of sociodemographic risk factors and measures of nicotine dependence in predicting maternal smoking during pregnancy. Drug Alcohol Depend..

[39] Mardby A.C., Lupattelli A., Hensing G., Nordeng H. (2017). Consumption of alcohol during pregnancy-A multinational European study. Women Birth.

[40] Corrales-Gutierrez I., Mendoza R., Gomez-Baya D., Leon-Larios F. (2020). Understanding the Relationship between Predictors of Alcohol Consumption in Pregnancy: Towards Effective Prevention of FASD. Int. J. Environ. Res. Public Health.

[41] Hamadneh S., Hamadneh J., Alhenawi E., Khurma R.A., Hussien A.G. (2024). Predictive factors and adverse perinatal outcomes associated with maternal smoking status. Sci. Rep..

[42] Azagba S., Ebling T., Korkmaz A. (2024). The Changing Faces of Smoking: Sociodemographic Trends in Cigarette Use in the U.S., 1992–2019. Int. J. Ment. Health Addict..

[43] Bover Manderski M.T., Steinberg M.B., Wackowski O.A., Singh B., Young W.J., Delnevo C.D. (2021). Persistent Misperceptions about Nicotine among US Physicians: Results from a Randomized Survey Experiment. Int. J. Environ. Res. Public Health.

[44] Gisle L., Demararest S., Drieskens S. (2018). Health Interview Survey 2018: Alcohol Use.

[45] Gisle L., Demararest S., Drieskens S. (2018). Health Interview Survery 2018: Smoking.

[46] Denny C.H., Acero C.S., Naimi T.S., Kim S.Y. (2019). Consumption of Alcohol Beverages and Binge Drinking Among Pregnant Women Aged 18–44 Years—United States, 2015–2017. Morb. Mortal. Wkly. Rep..

[47] Harrison P.A., Sidebottom A.C. (2009). Alcohol and drug use before and during pregnancy: An examination of use patterns and predictors of cessation. Matern. Child Health J..

[48] Liao S., Luo B., Feng X., Yin Y., Yang Y., Jing W. (2015). Substance use and self-medication during pregnancy and associations with socio-demographic data: A cross-sectional survey. Int. J. Nurs. Sci..

[49] Haight S.C., King B.A., Bombard J.M., Coy K.C., Ferre C.D., Grant A.M., Ko J.Y. (2021). Frequency of cannabis use during pregnancy and adverse infant outcomes, by cigarette smoking status—8 PRAMS states, 2017. Drug Alcohol Depend..

[50] Cerda M., Wall M., Keyes K.M., Galea S., Hasin D. (2012). Medical marijuana laws in 50 states: Investigating the relationship between state legalization of medical marijuana and marijuana use, abuse and dependence. Drug Alcohol Depend..

[51] Ishitsuka K., Hanada-Yamamoto K., Mezawa H., Saito-Abe M., Konishi M., Ohya Y., Japan E., Children’s Study G. (2020). Determinants of Alcohol Consumption in Women Before and After Awareness of Conception. Matern. Child Health J..

[52] Grittner U., Kuntsche S., Gmel G., Bloomfield K. (2013). Alcohol consumption and social inequality at the individual and country levels--results from an international study. Eur. J. Public Health.

[53] Goemaes R., Fomenko E., Laubach M., De Coen K., Roelens K., Bogaerts A. (2024). Perinatal Health in Flenders—Year 2023.

[54] Statistics Flanders Population and Society. https://www.vlaanderen.be/statistiek-vlaanderen/bevolking-en-samenleving.

[55] Growing Up, Child and Youth Govermental Agency Numerical Report Household Income and Poverty. https://www.opgroeien.be/kennis/cijfers-en-onderzoek/gezinsinkomen-en-kansarmoede.

[56] Statbel Employment and Unemployment. https://statbel.fgov.be/en/themes/work-training/labour-market/employment-and-unemployment.

[57] Backhausen M.G., Ekstrand M., Tyden T., Magnussen B.K., Shawe J., Stern J., Hegaard H.K. (2014). Pregnancy planning and lifestyle prior to conception and during early pregnancy among Danish women. Eur. J. Contracept. Reprod Health Care.

[58] Goossens J., Beeckman D., Van Hecke A., Delbaere I., Verhaeghe S. (2018). Preconception lifestyle changes in women with planned pregnancies. Midwifery.

[59] Goossens J., Delbaere I., Dhaenens C., Willems L., Van Hecke A., Verhaeghe S., Beeckman D. (2016). Preconception-related needs of reproductive-aged women. Midwifery.

[60] Amuli K., Decabooter K., Talrich F., Renders A., Beeckman K. (2021). Born in Brussels screening tool: The development of a screening tool measuring antenatal psychosocial vulnerability. BMC Public Health.

[61] Born in Belgium Professionals Born in Belgium Professionals. https://borninbelgiumpro.be/.

