# Supplementary material for: Alcohol, Tobacco and Illicit Drug Use During Pregnancy in the Longitudinal BELpREG Cohort in Belgium Between 2022 and 2024
Source: J Clin Med. 2025 Jan 18;14(2):613. doi: 10.3390/jcm14020613 (PMC11765680; doi:10.3390/jcm14020613)
Supplement: Supplementary file 1 [file jcm-14-00613-s001.zip › Supplementary Material 2.pdf]

Supplementary Material 2

Overview of the BELpREG data variables used in the analysis of alcohol, tobacco and illicit drug use

Overview of the BELpREG data variables, shown per category and indicating in which survey instruments they are asked (i.e., time points at which the information is collected; EM = Pregnancy Enrolment Questionnaire; FU = Pregnancy Follow-up Questionnaire; PP 1 = First Postpartum Questionnaire; PP 2 = Second Postpartum Questionnaire). This overview follows, where possible, the order of the variables as they appear in the BELpREG questionnaires.

**Category 1: Sociodemographics**								
**Variable**	**Definition**	**Values**	**Field type**	**Source**	**Survey Instruments**			
					**EM**	**FU**	**PP 1**	**PP 2**
BELpREG record id	Unique numeric code	Integer (Automatically assigned)	Text box	Automatically assigned	x	x	x	x
Date of birth	Date of birth participant	Date (DD-MM-YYYY)	Text box	Reported	x			
Age	Age of the participant at first day of the LMP[1]	Integer	Calculated field	*Derived*				
Partner	Currently having a partner	Y/N	Radio buttons	Reported	x			
Ethnicity participant	Ethnicity of the participant	European; Maghreb; Rest of Africa; Near/Middle East; Pacific/Far East; Rest of Asia; North America; Central and South America; I don't know / I prefer not to say	Checkboxes, Multiple choice	Reported	x			
Highest educational level of participant	Highest educational degree obtained by participant	Primary education; Lower secondary education (certificate 2nd degree); Higher secondary education; Higher vocational education (HBO5); Professional Bachelor / Higher education short type (non-university); Academic Bachelor; Master; PhD; Other (specify)	Radio buttons	Reported	x			
Highest educational level of partner	Highest educational degree obtained by current partner	Primary education; Lower secondary education (certificate 2nd degree); Higher secondary education; Higher vocational education (HBO5); Professional Bachelor / Higher education short type (non-	Radio buttons	Reported	x			

		university); Academic Bachelor; Master; PhD; Other (specify)						
Professional activity participant	The participant being professional active in the past year	Y/N	Radio buttons	Reported	x			
Gross Annual Family Income	Gross Annual Family Income	<15 000 euro; 15 000 euro - <30 000 euro; 30 000 euro - <45 000 euro; 45 000 euro - <65 000 euro; >=65 000 euro; I don't know / prefer not to say	Radio buttons	Reported	x			

Abbreviations : EM = Pregnancy Enrolment Questionnaire; FU = Pregnancy Follow-up Questionnaire; PP 1 = First Postpartum Questionnaire; PP 2 = Second Postpartum Questionnaire
Notes: [1] In case the last menstrual period (LMP) is not directly registered by participants, it is derived from estimated date of delivery (EDD).

**Category 2: Current pregnancy and health status**								
**Variable**	**Definition**	**Values**	**Field type**	**Source**	**Survey Instruments**			
					**EM**	**FU**	**PP 1**	**PP 2**
Estimated date of delivery (EDD)	Estimated date of delivery	Date (D-M-Y) and 'I don't know yet' checkbox	Text box and Checkbox	Reported	x		x	
Last Menstrual Period (LMP)	Date of the first day of the last menstrual period prior to conception	Date (D-M-Y)	Text box	Reported	x			
Onset of pregnancy	Onset of the current pregnancy	Spontaneously; After fertility treatment with hormonal stimulation; After fertility treatment without hormonal stimulation	Radio buttons	Reported	x			
Planned pregnancy	Planned pregnancy	Y/N	Radio buttons	Reported	x			
Willingness to breastfeed	Willingness to breastfeed	Y; N; I don't know	Radio buttons	Reported	x			
Length	Length at conception, in centimeter	Number	Text box	Reported	x			
Weight	Weight at conception, in kilogram	Number	Text box	Reported	x			
Body-Mass index (BMI)	BMI, calculated based on length and weight	Number	Calculated field	Derived				
Chronic conditions	Chronic conditions (i.e., conditions existing before the start of the pregnancy)	Text - selection from drop-down list[4] or free text - multiple answers possible	Drop-down list linked to database, Autocomplete	Reported	x			

Abbreviations : EM = Pregnancy Enrolment Questionnaire; FU = Pregnancy Follow-up Questionnaire; PP 1 = First Postpartum Questionnaire; PP 2 = Second Postpartum Questionnaire; AC = Auto Complete; MC = Multiple Choice; EDD = Estimated Date of Delivery; LMP = Last Menstrual Period; HCPs = Healthcare professionals; BMI = Body-Mass Index;

Category 3: Maternal-obstetric history								
**Variable**	**Definition**	**Values**	**Field type**	**Source**	**Survey Instruments**			
					**EM**	**FU**	**PP 1**	**PP 2**
Previous pregnancy	Previously having been pregnant	Y/N	Radio buttons	Reported	x			
Number of previous pregnancies	Number of previous pregnancies	Integer	Text box	Reported	x			
Gravidity	Gravidity	Primigravida; Multigravida	Calculated field	Derived				
Previous elective termination of pregnancy (ETOP)	Previous ETOP	Y/N	Radio buttons	Reported	x			

Abbreviations : EM = Pregnancy Enrolment Questionnaire; FU = Pregnancy Follow-up Questionnaire; PP 1 = First Postpartum Questionnaire; PP 2 = Second Postpartum Questionnaire; ETOP = Elective Termination Of Pregnancy; HELLP = Haemolysis, Elevated Liver enzymes and Low Platelets.

Category 5: Substance use								
**Variable**	**Definition**	**Values**	**Field type**	**Source**	**Survey Instruments**			
					**EM**	**FU**	**PP 1**	**PP 2**
**Tobacco use**								
Tobacco use - past year	Tobacco use in the past year	Y/N	Radio buttons	Reported	x			
Tobacco use - in pregnancy	Tobacco use during this pregnancy	Y/N	Radio buttons	Reported	x			
Timing of tobacco use cessation	Timing of tobacco use cessation	In the past year; Since trying to get pregnant; Since I knew I was pregnant	Radio buttons	Reported	x			
Tobacco use status throughout pregnancy	Tobacco use status throughout pregnancy	Y/N	Radio buttons	Reported	x	x		
Timing of tobacco use cessation in pregnancy	Gestational age upon tobacco use cessation	Number	Text box	Reported	x	x		
Amount of tobacco use in pregnancy	Daily amount of tobacco use in this pregnancy	*Since start data collection:*<br>1-10 cigarettes/day; 11-20 cigarettes/day; 21-30 cigarettes/day; >30 cigarettes/day<br>*Since July 2024:*<br><1 cigarette/day; 1-5 cigarettes/day; 6-10 cigarettes/day; 11-20 cigarettes/day; >20 cigarettes /day	Radio buttons	Reported	x	x		
Exposure to second-hand tobacco use during pregnancy	Tobacco use by someone who lives together with the pregnant participant	Y/N	Radio buttons	Reported	x			

**Alcohol use**								
Alcohol use - past year	Use of alcohol in the past year	Y/N	Radio buttons	Reported	x			
Alcohol use - in pregnancy	Use of alcohol during this pregnancy	Y/N	Radio buttons	Reported	x			
Timing of alcohol cessation	Timing of alcohol cessation	In the past year; Since trying to get pregnant; Since I knew I was pregnant	Radio buttons	Reported	x			
Alcohol use status throughout pregnancy	Alcohol use status throughout pregnancy	Y/N	Radio buttons	Reported	x	x		
Timing of alcohol use cessation in pregnancy	Gestational age upon alcohol use cessation	Number	Text box	Reported	x	x		
Frequency of drinking alcohol in pregnancy	Frequency of drinking alcohol in this pregnancy	Monthly or less; 2-4 times a month; 2-3 times a week; 4 or more times a week	Radio buttons	Reported	x	x		
Amount of alcohol per occasion	Number of standard glasses of alcohol used per occasion. 1 standard glass is the amount of a drink that is usually served in a pub or at a restaurant, e.g., 25cl beer or 10cl wine	<1; 1 or 2; 3 or 4; 5 or 6; 7 or 9; 10 or more	Radio buttons	Reported	x	x		
Occurrence of binge drinking	Occurrence of using 6 or more standard glasses of alcohol per occasion	Never; Less than monthly; Monthly; Weekly; Daily or almost daily	Radio buttons	Reported	x	x		
Alcohol use in the close environment during pregnancy	Daily use of alcohol by someone who lives together with the pregnant participant	Y/N	Radio buttons	Reported	x			
**Cannabis and other illicit drugs**								
Cannabis and other illicit drug use - past year	Use of cannabis and other illicit drugs in the past year	Y/N	Radio buttons	Reported	x			
Cannabis and other illicit drug use - in pregnancy	Use of cannabis and other illicit drugs in this pregnancy	Y/N	Radio buttons	Reported	x			
Timing of cessation of use of cannabis and other illicit drugs	Timing of cessation of the use of cannabis and other illicit drugs	In the past year; Since trying to get pregnant; Since I knew I was pregnant	Radio buttons	Reported	x			
Type of drugs used	Type of drugs used in pregnancy or after delivery, i.e., cannabis (hasj, weed, marihuana), XTC (MDMA), amphetamines (speed,…), hallucinogens (LSD, magic mushrooms), cocaine, ketamine, heroine, GHB, other (specify)	Never used; Less than once a month; Multiple times a month; Once a week; Multiple times a week; Daily	Matrix with radio buttons	Reported	x	x		

Drug use in the close environment during pregnancy	Drug use by someone who lives together with the pregnant participant	Y/N	Radio buttons	Reported	x			

Abbreviations : EM = Pregnancy Enrolment Questionnaire ; FU = Pregnancy Follow-up Questionnaire ; PP 1 = First Postpartum Questionnaire ; PP 2 = Second Postpartum Questionnaire.
