# Supplementary material for: Alcohol, Tobacco and Illicit Drug Use During Pregnancy in the Longitudinal BELpREG Cohort in Belgium Between 2022 and 2024
Source: J Clin Med. 2025 Jan 18;14(2):613. doi: 10.3390/jcm14020613 (PMC11765680; doi:10.3390/jcm14020613)
Supplement: Supplementary file 1 [file jcm-14-00613-s001.zip › Supplementary Material 3.pdf]

Supplementary Material 3

Sensitivity analysis

Sensitivity analysis: multivariable logistic regression analyses for alcohol use and tobacco use in the 1st trimester using a stepwise approach

Table S3. Associations between maternal characteristics and alcohol use in the first trimester.

	Alcohol use during 1st trimester			
	**cOR**	**95% CI**	**aOR**	**95% CI**
Maternal age (in years)			-	-
18-24	1.34	0.44-4.09		
25-29	Reference			
30-34	1.00	0.69-1.46		
35-39	0.71	0.64-1.92		
>40	0.32	0.05-2.70		
Maternal ethnical background			-	-
Non-Caucasian or I don't know	0.56	0.07-4.31		
Marital status			-	-
No partner	0.21	0.03-1.56		
Maternal education			-	-
Low/ medium	0.94	0.57-1.55		
Maternal preconception BMI (kg/m^2)				
<18.5	0.71	0.25-2.05	0.85	0.29-2.52
18.5-25	Reference			
25-30	0.89	0.59-1.33	0.88	0.58-1.35
>30	0.60*	**0.35-1.05**	0.62	0.35-1.10
Chronic condition prior to pregnancy				
Yes	0.73	0.51-1.04	0.79	0.55-1.15
Planned pregnancy				
No	3.79*	**2.40-5.97**	2.63	**1.62-4.28**
Method of conception				
Not spontaneous	0.27*	**0.14-0.52**	0.35	**0.18-0.68**
Gravidity			-	-
Multigravida	0.98	0.70-1.37		
Previous planned abortion			-	-
Yes	1.35	0.73-2.51		
Cohabitant drinking alcohol daily				
Yes	2.14*	**1.19-3.84**	1.96	**1.06-3.63**
Cohabitant using tobacco			-	-
Yes	1.02	0.63-1.65		
Cohabitant using illicit drugs			-	-
Yes	1.57	0.64-3.87		
Preconception tobacco use				
Yes	2.28*	**1.45-3.59**	0.84	0.38-1.84
Illicit drug use in trimester 1				
Yes	17.39*	**3.34-90.40**	4.58	0.71-26.70

Tobacco use in trimester 1				
Yes	5.48*	**3.04-9.88**	3.89	**1.46-10.37**

*p-value < 0.1.
cOR = crude odds ratios; aOR = adjusted odds ratios, adjusted for maternal preconception BMI, chronic condition prior to pregnancy, planned pregnancy, method of conception, cohabitant drinking alcohol daily, preconception tobacco use, illicit drug use in trimester 1, tobacco use in trimester 1
Note: The bold numbers indicate adjusted odds ratios where the 95% confidence interval does not include 1.

Table S4. Associations between maternal characteristics and tobacco use in the first trimester.

	**Tobacco use during 1st trimester**			
	**cOR**	**95% CI**	**cOR**	**95% CI**
Maternal age (in years)				
18-24	3.53*	**9.48-13.16**	0.44	0.08-2.31
25-29	Reference			
30-34	0.81	0.41-1.60	1.08	0.46-2.54
35-39	1.99*	**0.89-4.43**	2.00	0.67-6.02
>40	1.30	0.16-10.38	2.98	0.27-32.70
Maternal ethnical background				
Non Caucasian or I don't know	4.38*	**0.95-20.32**	2.54	0.34-19.27
Marital status			-	-
No partner	2.51	0.74-8.53		
Maternal education				
Low/ medium	10.7*	**5.96-19.39**	9.38	**4.34-20.27**
Maternal preconception BMI (kg/m^2)			-	-
<18.5	0.69	0.09-5.19		
18.5-25	Reference			
25-30	1.29	0.67-2.49		
>30	1.20	0.54-2.69		
Chronic condition prior to pregnancy			-	-
Yes	1.0	0.60-1.90		
Planned pregnancy				
No	6.56*	**3.51-12.27**	3.05	**1.36-6.85**
Method of conception				
Not spontaneous	0.28*	**0.09-0.89**	0.37	0.10-1.39
Gravidity				
Multigravida	1.65*	**0.92-2.98**	0.97	0.42-2.23
Previous planned abortion				
Yes	3.45*	**1.61-7.38**	1.81	0.65-5.09
Cohabitant who drinks alcohol daily				
Yes	2.39*	**0.98-5.82**	0.97	0.32-2.97
Cohabitant using tobacco				
Yes	11.2*	**6.23-20.41**	9.30	**4.44-19.49**
Cohabitant using illicit drugs				
Yes	4.69*	**1.73-12.75**	0.51	0.11-2.67
Preconception alcohol use			-	-
Yes	1.22	0.57-2.64		
Illicit drug use in trimester 1				
Yes	159.96*	**18.85-1357.20**	28.96	**2.41-348.19**

Alcohol use in trimester 1				
Yes	5.48*	**3.04-9.88**	6.38	**2.92-13.97**

*p-value < 0.1

cOR = crude odds ratios; aOR = adjusted odds ratios, adjusted for maternal age, maternal ethnical background, maternal education, planned pregnancy, method of conception, gravidity, previous planned abortion, cohabitant who drinks alcohol daily, cohabitant using tobacco, cohabitant using illicit drugs, illicit drug use in trimester 1, alcohol use in trimester 1

Note: The bold numbers among crude odds ratios indicate a p-value < 0.10. Bold numbers among adjusted odds ratios indicate a 95% confidence interval where does not include 1.
