# Supplementary material for: Alcohol, Tobacco and Illicit Drug Use During Pregnancy in the Longitudinal BELpREG Cohort in Belgium Between 2022 and 2024
Source: J Clin Med. 2025 Jan 18;14(2):613. doi: 10.3390/jcm14020613 (PMC11765680; doi:10.3390/jcm14020613)
Supplement: Supplementary file 1 [file jcm-14-00613-s001.zip › Supplementary Material 4.pdf]

Supplementary Material 4

Sample comparison

Table S5. Comparison of characteristics between participants who had delivered and those who had not yet delivered or were lost to follow-up by the time of data extraction from the BELpREG pregnancy registry.

Characteristics	Participants who had delivered by the time of data extraction[1] (n=645)		Participants who had not delivered yet or were lost to follow-up (n=579)		Chi-square	
	N	%	N	%	X^2	p-value[2]
**Sociodemographic variables**						
Maternal age (in years)					4.83	0.31
18-24	14	2.2	10	1.7		
25-29	218	33.8	168	29.0		
30-34	328	50.9	305	52.7		
35-39	70	10.9	78	13.5		
>40	12	1.9	8	1.4		
Missing	3	0.5	10	1.7		
Maternal ethnical background					2.60	0.11
Caucasian	641	99.4	564	97.4		
Non-Caucasian or I don't know	4	0.6	9	1.6		
Missing	0	0.0	6	1.0		
Marital status					0.00	0.98
Partner	628	96.4	558	96.4		
No partner	17	2.6	15	2.6		
Missing	0	0.0	6	1.0		
Maternal education[3]					0.51	0.48
Low/ medium	90	14.0	72	12.4		
High	555	86.0	501	86.5		
Missing	0	0.0	6	1.0		
Paternal education[3]					0.09	0.77
Low/ medium	201	32.0	175	31.4		
High	423	67.4	382	68.5		
Missing	4	0.6	1	0.2		
Maternal employment status in the past year					0.17	0.68
Employed	628	97.4	560	96.7		
Not employed	17	2.6	13	2.2		
Missing	0	0.0	6	1.0		
Household annual gross income					2.46	0.48
<45.000 euro	85	13.2	66	11.4		
45.000 – 65.000 euro	155	24.0	136	23.5		
>65.000 euro	242	37.5	238	41.1		
I don't know/ I rather not tell	163	25.3	133	23.0		
Missing	0	0.0	6	1.0		
Maternal preconception BMI (kg/m^2)					1.44	0.70
<18.5	23	3.6	15	2.6		

18.5-25	380	58.9	332	57.3		
25-30	153	23.7	137	23.7		
>30	89	13.8	88	15.2		
Missing	0	0.0	7	1.2		
Chronic medical condition prior to	399	38.1	224	38.7	0.12	0.73
pregnancy[4]	246	61.9	349	60.3		
Yes	0	0.0	6	1.0		
No						
Missing						
**Pregnancy related variables**						
Planned pregnancy					0.00	1.00
Yes	591	91.6	525	90.7		
No	54	8.4	48	8.3		
Missing	0	0.0	6	1.0		
Method of conception					0.08	0.78
Spontaneous	525	81.4	470	81.2		
ART[5]	120	18.6	103	17.8		
Missing	0	0.0	6	1.0		
Gravidity					0.09	0.76
Primigravida	305	47.3	276	47.7		
Multigravida	340	52.7	297	51.3		
Missing	0	0.0	6	99.0		
Previous planned termination of pregnancy					1.19	0.28
Yes	37	10.9	42	14.1		
No	301	88.5	255	85.9		
Missing	2	0.6	0	0.0		
Intention to breastfeed					0.53	0.47
Yes	549	85.1	479	82.7		
No or I don’t know yet	96	14.9	94	16.2		
Missing	0	0.0	6	1.0		
**Social/environmental variables**						
Cohabitant who was drinking alcohol daily					0.38	0.54
Yes	39	6.0	30	5.2		
No	605	93.9	543	93.8		
Missing	1	0.2	6	1.0		
Cohabitant who was tobacco use					1.13	0.29
Yes	82	12.7	85	14.7		
No	562	87.1	488	84.3		
Missing	1	0.2	6	1.0		
Cohabitant who was using illicit drugs					0.00	0.98
Yes	17	2.6	15	2.6		
No	627	97.2	558	96.4		
Missing	1	0.2	6	1.0		

[1] Considering all women who had given birth at the time of data extraction, except for women who did not provide data on substance use in the enrolment questionnaire.
[2] p-value ≤ 0.05.
[3] High education = any formal degree obtained in higher education or university.
[4] A chronic condition prior to pregnancy was defined as any diagnosed chronic condition before the start of the index pregnancy.
[5] ART is defined as assisted reproductive technology.
